# A Fuel‐Driven Lock‐and‐Key System

**DOI:** 10.1002/open.202500042

**Published:** 2025-06-09

**Authors:** Shilin Zhang, Yanan Zhu, Hailiang Ni, Ping Hu, Yibin Sun

**Affiliations:** ^1^ College of Chemistry and Materials Science Sichuan Normal University Chengdu 610068 P. R. China; ^2^ Faculty of Materials Science Shenzhen MSU‐BIT University Shenzhen 518172 P. R. China; ^3^ College of Chemistry and Molecular Engineering Peking University Beijing 100871 P. R. China

**Keywords:** chemical fuel, crown ether, host‐guest, lock‐and‐key

## Abstract

This study introduces a fuel‐driven lock‐and‐key system based on the interaction between crown ether and ammonium ion. In this simple model system, a key‐like molecule with an amino group functions as the key, while 15‐crown‐5 serves as the lock. The chemical fuel, 2‐cyano‐2‐phenylpropanoic acid, protonates the key, transitioning it from its deprotonated state to a protonated state, enabling it to bind to the lock. Upon fuel consumption, the protonated key reverts to its deprotonated state, causing the dissociation from the lock. This cycle is reversible and can be repeated at least three times. We hope that this intuitive lock‐and‐key system can provide a clearer understanding of energy‐driven molecular recognition and offer valuable insights into the design and development of energy‐driven molecular systems based on molecular recognition.

## Graphical abstract

A fuel‐driven lock‐and‐key system is based on crown ether (L) and ammonium ions, showing reversible binding between deprotonated key (D‐K) and protonated key (P‐K). Energy input drives D‐K to P‐K, while fuel consumption reverses the process, enabling controlled molecular recognition and state transitions.

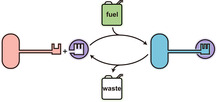

## 1 Introduction

The lock‐and‐key model, proposed by Emil Fischer in 1894,^[^ ^1^ ^]^ is a pivotal concept in understanding molecular recognition.^[^ ^2^, ^3^, ^4^, ^5^ ^]^ Initially describing how enzymes interact with substrates in biological systems, the model has since evolved into a fundamental framework for studying selectivity and specificity in molecular interactions, both in biological processes and synthetic supramolecular systems.^[^ ^5^, ^6^, ^7^, ^8^, ^9^, ^10^, ^11^, ^12^ ^]^ This principle of precise complementarity between a host molecule (the lock) and a guest molecule (the key) has guided the design of numerous molecular machines and functional supramolecular systems, based on the intelligent design of switchable binding sites between host and guest components.^[^ ^13^, ^14^, ^15^, ^16^, ^17^, ^18^, ^19^, ^20^, ^21^, ^22^, ^23^ ^]^

In the operation of molecular machines, repeated energy inputs are often required to drive the system's transitions.^[^ ^24^, ^25^, ^26^, ^27^, ^28^, ^29^, ^30^, ^31^, ^32^, ^33^ ^]^ These inputs, such as chemical fuels or external stimuli,^[^ ^34^, ^35^, ^36^, ^37^ ^]^ enable the reversible interactions between lock‐and‐key motifs, allowing precise control over the machine's state transitions.^[^ ^38^, ^39^, ^40^, ^41^ ^]^ The development of energy‐responsive host–guest systems is thus crucial for constructing advanced molecular machines and supramolecular systems with applications in drug delivery, chemical sensing, and adaptive materials.

In this context, we report a simple, fuel‐driven lock‐and‐key system based on the interaction between crown ethers and ammonium ions. This system not only exemplifies the lock‐and‐key model but also serves as a basic platform to illustrate the fundamental principles of molecular recognition in energy‐driven systems. To enhance the clarity of the concept, we designed the key molecule to resemble the shape of a physical key, thereby making the model system intuitive for understanding the relationship between molecular recognition and energy‐driven processes.

## 2 Results and Discussion

**Figure 1:**
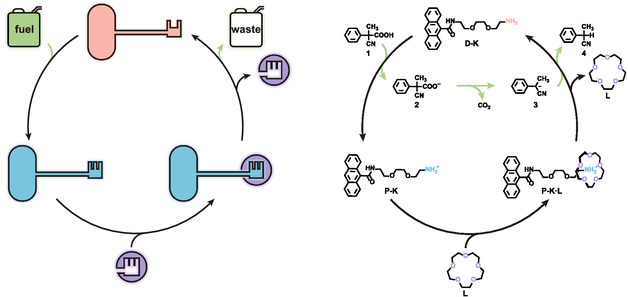
Schematic illustration of the fuel‐driven lock‐and‐key system: (left) cartoon representation showing the conceptual operation of the system and (right) molecular structures detailing the components involved.

The design of the fuel‐driven lock‐and‐key system is illustrated in **Figure** **1**. The lock (**L**) molecule selected for this system is 15‐crown‐5, a well‐studied macrocycle known for its selective binding to protonated amines with binding constants typically on the order of 10^2^ M^−1^,^[^ ^42^ ^]^ while not interacting with amino groups. This selectivity facilitates the overall design of the system. Additionally, the ^1^H NMR spectrum of 15‐crown‐5 displays a single peak, which simplifies the analysis of its binding interactions with the guest molecule using NMR spectroscopy.

The key motif is designed with an anthracenyl group as the key head, complemented by an amino group for key notches, all connected through an amide bond and an alkoxy chain that form the key neck. Initially, this key is referred to as deprotonated key (**D‐K**), and it cannot bind to the lock on its own. Upon protonation, it transforms into a protonated key (**P‐K**), allowing it to bind effectively to the lock.

The chemical fuel used in this system is 2‐cyano‐2‐phenylpropanoic acid (**1**), an activated carboxylic acid widely recognized for its role in driving various molecular machines.^[^ ^43^, ^44^ ^]^ Initially, this fuel acts as an acid, protonating the key and enabling its binding to the lock. After binding, the fuel undergoes decarboxylation to form a benzyl anion. This benzyl anion, which is a strong base, then abstracts a proton from the protonated key, returning it to deprotonated state.

**Figure 2:**
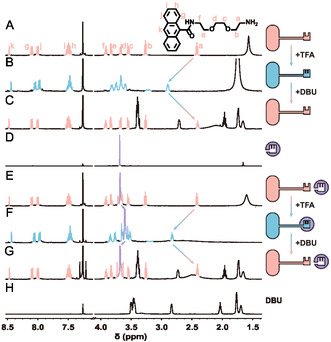
Monitoring each state of the lock‐and key system using ^1^H NMR spectroscopy. To investigate the protonated and deprotonated states of the key, 3 mM **D‐K** was first dissolved in A) CDCl_3_, followed by the addition of B) 3 mM TFA, and then an excess of C) DBU. D) shows the ^1^H NMR spectrum of **L**. To study the binding and dissociation of the lock and key, 3 mM **L** was dissolved in CDCl_3_, then E) 3 mM **D‐K** was added, followed by F) 3 mM TFA, and finally an excess of G) DBU. H) shows the ^1^H NMR spectrum of DBU alone.

The synthetic procedure for **D‐K** is outlined in Figure S1, Supporting Information, together with its ^1^H NMR spectrum (**Figure** **2**A, Figure S2, Supporting Information), ^13^C NMR (Figure S3, Supporting Information), and ^1^H–^1^H COSY (Figure S4, Supporting Information) spectra for structural confirmation. With **D‐K** synthesized, we first utilized trifluoroacetic acid (TFA) and diazabicycloundecene (DBU) to toggle the key between its deprotonated and protonated state, and to assess whether **P‐K** could effectively bind to **L**.

Upon adding one equivalent of TFA, the signal for *H*_a_—the protons on the methylene group attached to the amino group—shifts from 2.42 to 2.89 ppm (Figure 2A,B), indicating the protonation of the amino group to form an ammonium ion. In addition to *H*_a_, the *H*_k_ proton on the anthracene moiety also exhibits a detectable chemical shift change during the protonation process, providing an additional indicator for distinguishing between **D‐K** and **P‐K** states (see Figure S5, Supporting Information). Further addition of n equivalents of DBU leads to the complete deprotonation of the ammonium ion, causing the *H*_a_ signal to revert to 2.41 ppm (Figure 2C). This reversible chemical shift serves as a reliable indicator of the conversion between **D‐K** and **P‐K**.

When **D‐K** is mixed with **L** in equimolar amounts in CDCl_3_, the resulting NMR spectrum reveals a simple superposition of signals from both compounds, confirming that **D‐K** does not bind to **L** (Figure 2D,E). Specifically, the protons in **L** appear as a singlet around 3.68 ppm. Upon the addition of an equivalent of TFA, **D‐K** is transformed into **P‐K**, which is evidenced by a downfield shift of *H*_a_ to 2.88 ppm and an upfield shift of **L**'s peak to 3.60 ppm (Figure 2F). Moreover, the *H*_a_ proton signal exhibits clear fine structures, distinct from the broad peak observed in the protonated key alone (Figure 2B), indicating a change in the chemical environment upon complexation. The shift of **L**'s proton signal by 0.08 ppm is also significant, considering that typical chemical shift changes for crown ether‐ammonium binding are reported to be around 0.05 ppm.^[^ ^45^ ^]^ These observations collectively confirm the successful formation of a host–guest complex between **P‐K** and **L**.

The subsequent addition of excess DBU facilitates the reversion of **P‐K** to **D‐K**, accompanied by the dissociation from **L**. This transition is illustrated in Figure 2G, where all peaks corresponding to **D‐K** and **L** return to their original chemical shifts. This observation confirms that the binding and dissociation of the key and lock can be effectively regulated through acid‐base conditions. Moreover, we have identified specific peaks in the NMR spectrum that indicate the conversion between **D‐K** and **P‐K**, as well as the binding interaction between **L** and the key. Full spectra of Figure 2 are provided in Figure S6, Supporting Information.

Additionally, we explored the possibility of monitoring this process using ultraviolet‐visible and fluorescence spectroscopy. However, the transition from **P‐K** to **D‐K** only produced a slight decrease in spectral intensity, as detailed in Figure S7, Supporting Information. The corresponding theoretical calculations provided insights into the reason for the minimal spectral changes, as shown in Figure S8–S11 and Table S1,S2, Supporting Information. Consequently, to accurately monitor and represent the various states within the system, we opted for ^1^H NMR spectroscopy as our primary characterization technique.

**Figure 3:**
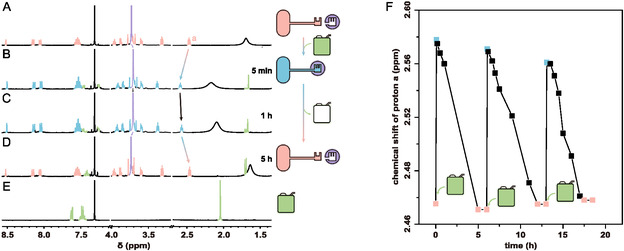
Monitoring the fuel‐driven binding between lock and key using ^1^H NMR spectroscopy. To complete one cycle, 3 mM **D‐K** and 3 mM **L** were dissolved in A) CDCl_3_, followed by the addition of B) 3 mM **1**. Spectra were recorded after C) 1 h and D) 5 h. E) shows the ^1^H NMR spectrum of **1** alone. The cycle was repeated three times, with an additional 3 mM of **1** added each time, and the F) chemical shift of *H*_a_ was monitored over time.

Following confirmation that the binding between key and lock can be controlled by acid‐base conditions and that the different states can be identified using ^1^H NMR spectroscopy, we proceeded to implement the design outlined in Figure 1. In this experiment, we utilized **1** as a chemical fuel to drive the fuel‐driven binding of the lock and key, followed by active separation upon the consumption of chemical fuel (**Figure** **3**A–E, and full spectra of are provided in Figure S12, Supporting Information).

To initiate the process, equimolar amounts of **D‐K** and **L** were mixed in CDCl_3_ (Figure 3A), and an equivalent amount of **1** was added. Within 5 min, **D‐K** rapidly converted to **P‐K** and bound to **L**, as evidenced by the corresponding shifts in the ^1^H NMR peaks (Figure 3B). The chemical shift of *H*_a_ under fuel‐driven conditions (2.58 ppm) is lower than that observed upon direct TFA protonation (2.89 ppm), which can be attributed to differences in acidity: The chemical fuel **1** has an estimated p*K*_*a*_ of ≈2.37, while TFA has a much lower p*K*_*a*_ of ≈0.23. The weaker acidity of the fuel leads to a lower degree of protonation, resulting in a smaller downfield shift. Similar phenomena have been commonly observed in fuel‐driven host–guest systems,^[^ ^44^, ^46^ ^]^ and the partial protonation here does not affect the successful binding and reversible operation of the system.

Concurrently, all the chemical fuel **1** underwent deprotonation, forming compound **2**. The most notable change was the shift of the methyl peak of **1** from 2.15 to 1.68 ppm, indicating the conversion, along with the appearance of weak singlets at 1.70 ppm, which correspond to the methyl groups of waste product (waste **4**).

As the chemical fuel was consumed, **P‐K** gradually underwent deprotonation, reverting to **D‐K** (Figure 3C,D). After 5 h, the decarboxylation of **2** to **4** was confirmed by the gradual decrease in the 1.68 ppm singlet, which eventually disappeared, while the 1.70 ppm peak intensified. Simultaneously, the key completely detached from the lock, along with the depletion of the fuel.

Notably, the chemical fuel was nearly entirely directed toward protonating the key rather than undergoing self‐degradation in this cycle. This conclusion was supported by control experiments, where a solution containing the same concentration of the chemical fuel remained stable for 30 h without significant decomposition (Figure S13, Supporting Information).

The fuel‐driven cycling capability of this system was demonstrated through three consecutive cycles using the same sample. In each cycle, addition of one equivalent of fuel triggered the transformation from **D‐K** to **P‐K**, as indicated by the characteristic chemical shift change (from 2.47 ppm to 2.56–2.57 ppm). As the fuel was consumed, the system consistently returned to the D‐K state, completing each cycle (Figure 3F). This reversible cycling behavior is consistent with other fuel‐driven molecular systems reported in recent studies,^[^ ^47^, ^48^, ^49^, ^50^, ^51^, ^52^, ^53^, ^54^ ^]^ where similar multiple‐cycle operations have been demonstrated. The successful execution of three complete cycles confirms the robustness and reproducibility of our fuel‐driven system.

## 3 Conclusion

In conclusion, we present a simple yet reversible lock‐and‐key system driven by a chemical fuel. By utilizing ^1^H NMR spectroscopy, we demonstrated the fuel‐driven binding and dissociation of the key from the lock, offering a controllable mechanism for state transitions. The system's reversible nature, observed across multiple cycles, suggests its potential application in constructing more complex energy‐driven molecular systems, such as advanced molecular machines and functional supramolecular systems.

Moreover, by designing the key molecule to resemble a physical key, we provide an intuitive model that integrates molecular recognition with energy‐driven processes. This system serves as an accessible platform for understanding fundamental concepts: 1) the lock‐and‐key principle, 2) fuel‐driven molecular transformations, and 3) their experimental characterization. The macroscopic key‐lock analogy makes these concepts more tangible, particularly for beginning researchers. We hope this work advances our understanding of fuel‐driven systems while serving as an effective educational tool in supramolecular chemistry.

## Conflict of Interest

The authors declare no conflict of interest.

## Supporting information

Supplementary Material

## Data Availability

The data that support the findings of this study are available in the supplementary material of this article.
