# Supplementary material for: A Fuel‐Driven Lock‐and‐Key System
Source: ChemistryOpen. 2025 Jun 9;14(9):e202500042. doi: 10.1002/open.202500042 (PMC12409834; doi:10.1002/open.202500042)
Supplement: Supplementary file 1 — Supplementary Material [file OPEN-14-e202500042-s001.pdf]

Supporting Information for

A Fuel-Driven Lock-and-Key System

Shilin Zhang[a,1], Yanan Zhu[b,1], Hailiang Ni[a], Ping Hu[a], and Yibin Sun[c,*]

[a] *College of Chemistry and Materials Science, Sichuan Normal University, Chengdu 610068, P. R. China.*
[b] *Faculty of Materials Science, Shenzhen MSU-BIT University, Shenzhen, 518172, P.R. China.*
[c] *College of Chemistry and Molecular Engineering, Peking University, Beijing 100871, P. R. China.*
[*] *e-mail: sunyibin2011@pku.edu.cn*
[1] *These authors contributed equally to this work.*

Contents

1 Supplementary Methods **2**

2 Supplementary Figures and Tables **4**

1 Supplementary Methods

Materials

All reagents were commercially available from Alfa, Sigma Aldrich and TCI, and used without further purification.

^{1}H nuclear magnetic resonance (NMR) spectra were recorded on a Bruker AVIII-500 spectrometer (500 MHz, 298 K) and were reported relative to residual solvent signals.

UV-vis spectra were collected on SHIMADZU UV-2700. Fluorescence spectra were collected on HITACHI F-4500.

Theoretical Calculations

The geometric optimization of studied molecules at ground states are performed with B3LYP-D3BJ[1, 2] and 6-31G(d,p) basis set[3], which includes dispersion interactions. The single point energy including frontier molecular orbitals and electrostatic potential (ESP) are calculated at DEF2TZVP basis set[4, 5] for achieving higher accuracy. For excited states properties, the vertical excitation energy and adiabatic excitation energy of S1 based on converged geometries are obtained by M062X with DEF2TZVP, where the geometric structure of excited states are optimized by the 2-zeta basis set with the same functional for better cost-efficiency. All the computations are performed with Gaussian 09 D.01 version and the analyzation of data is assisted with Multiwfn[6, 7] and VMD 1.9.3[8].

Synthetic Procedures

Synthesis of 1. Chemical fuel 1 was synthesized according to the literature mothod[9].

In a 100 mL Schlenk flask, ethyl cyanoacetate (2.15 mL, 20 mmol), iodobenzene (1.15 mL, 10 mmol), potassium carbonate (5.5 g, 40 mmol), and DMSO (30 mL) were sequentially added. Under a nitrogen atmosphere, cuprous iodide (0.19 g, 1 mmol) was then added to the reaction mixture. The mixture was stirred at 120 °C for 20 hours to complete the reaction. After cooling to room temperature, iodomethane (2.5 mL, 40 mmol) was added to the brown mixture under a nitrogen atmosphere. The reaction was continued at room temperature for 10 hours. The reaction mixture was then transferred into dilute hydrochloric acid and extracted with ether. The organic phase was dried over anhydrous sodium sulfate, followed by filtration and evaporation of the organic solvent under reduced pressure to obtain a solid mixture. The mixture was purified by column chromatography with dichloromethane:petroleum ether = 1:5 (v/v) to give **1-Et** as liquid (1.27 g, 67.8%). ^{1}H NMR (500 MHz, $CDCl_3$, ppm): δ 7.54–7.51 (m, 2H), 7.44–7.35 (m, 3H), 4.30–4.18 (m, 2H), 1.96 (s, 3H), 1.25 (t, 3H).

1-Et (1 g, 4.92 mmol) was added to a round-bottom flask containing ethanol (10 mL), followed by the addition of a potassium hydroxide solution (0.825 g, 14.7 mmol) in water (10 mL). The mixture was stirred at room temperature for 20 hours, and the reaction was stopped when TLC monitoring indicated no starting material remained. Ethanol was removed under reduced pressure, and 20 mL of water was added to the mixture. The mixture was then acidified with dilute sulfuric acid under an ice bath, and extraction was performed with ether. The ether phases were combined and dried over anhydrous sodium sulfate, followed by filtration. The organic solvent was removed under reduced pressure at room temperature to yield an oily liquid. Hexane was added to the mixture, which was then placed in a −20 °C freezer. After the formation of a white solid, filtration was performed, and the solid was dried to obtain a white solid (0.31 g, 36.0%). ^{1}H NMR (500 MHz, $CDCl_3$, ppm): δ 7.58–7.56 (m, 2H), 7.46–7.39 (m, 3H), 1.99 (s, 3H). ^{1}3C NMR (125 MHz, $CDCl_3$, ppm): δ 173.21, 134.68, 129.33, 129.27, 125.95, 118.88, 48.33, 24.21.

Synthesis of D-K.

1,2-Bis(2-aminoethoxy)ethane (9.8 mL, 67.5 mmol) and hexafluorophosphate benzotriazol-1-yl-oxy-tripyrrolidinophosphine (PyBOP) (8.2 g, 17.6 mmol) were added to a round-bottom flask containing 30 mL of dichloromethane. A solution of 9-anthracene carboxylic acid (3 g, 13.5 mmol) in DMSO (5 mL) was then added. The orange-yellow solution was stirred at room temperature overnight under a nitrogen atmosphere. The reaction was stopped when TLC monitoring showed no starting material (9-anthracene carboxylic acid) remained. Extraction was carried out using a dichloromethane/water system, and the organic phase was combined and washed with saturated saline solution. The organic phase was dried over anhydrous sodium sulfate, filtered, and the solvent was removed under reduced pressure to yield the crude product. The crude product was purified by column chromatography with dichloromethane:methanol = 15:1 (v/v) to give **D-K** as light brown solid (3.06 g, 64.3%). ^{1}H NMR (500 MHz, $CDCl_3$, ppm): δ 8.47 (s, 1H), 8.10 (d, 2H), 8.00 (d, 2H), 7.52–7.46 (m, 4H), 7.28 (s, 1H), 3.91 (q, 2H), 3.82 (dd, 2H), 3.67–3.66 (m, 2H), 3.55–3.53 (m, 2H), 3.26 (t, 2H), 2.41 (t, 2H). ^{1}3C NMR (125 MHz, $CDCl_3$, ppm): δ 169.65, 132.17, 131.07, 128.46, 128.04, 128.02, 126.57, 125.49, 125.25, 72.71, 70.41, 69.95, 69.86, 41.14, 39.86.

2 Supplementary Figures and Tables

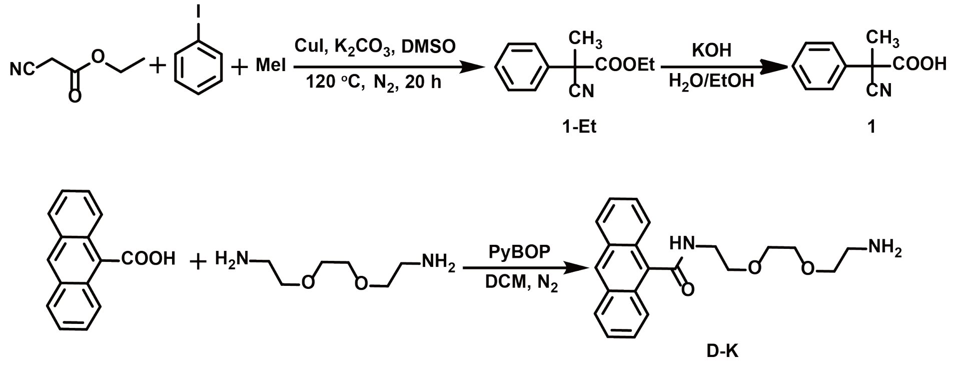

Figure S1: Synthetic procedures of **1** and **D-K**.

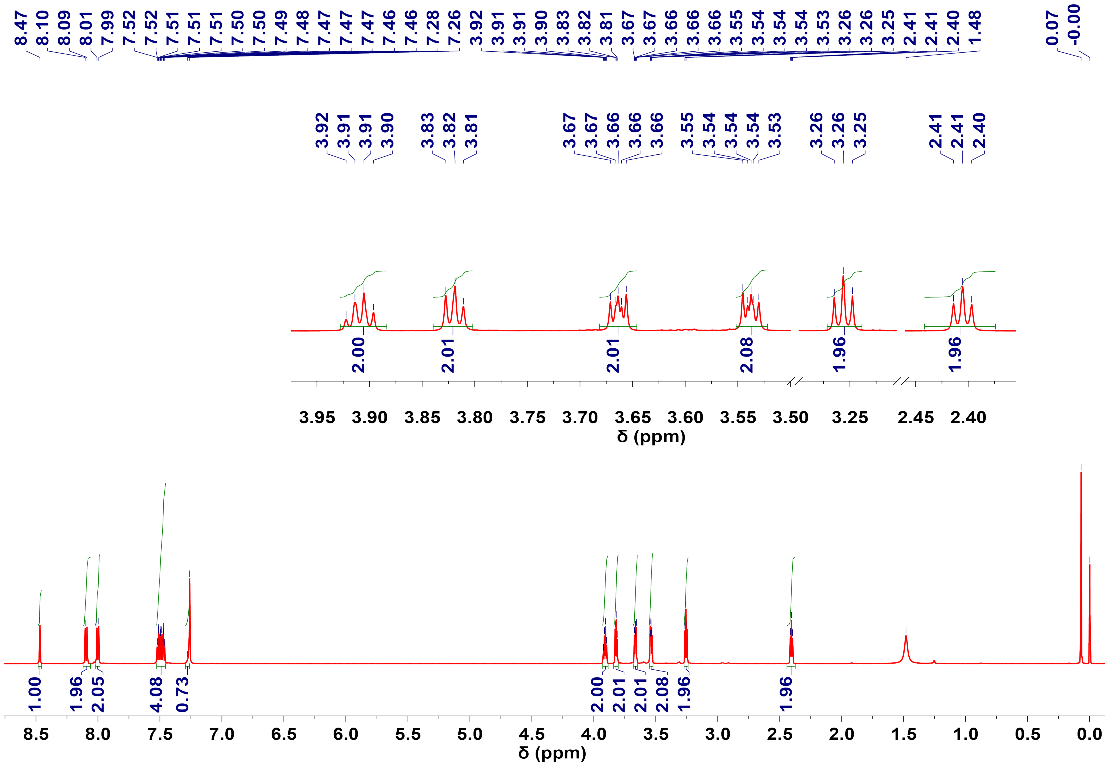

Figure S2: ^1H NMR (500 MHz, $CDCl_3$) spectrum of **D-K**.

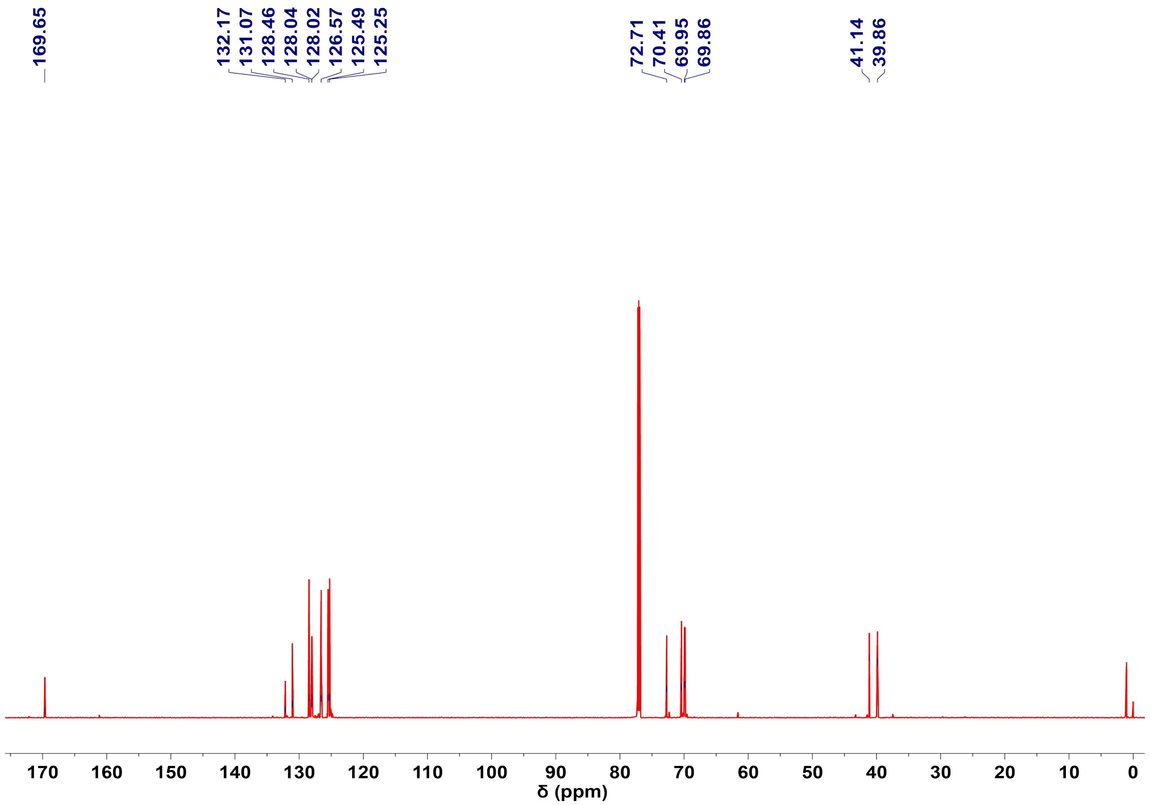

Figure S3: ^{13}C NMR (125 MHz, $CDCl_3$) spectrum of **D-K**.

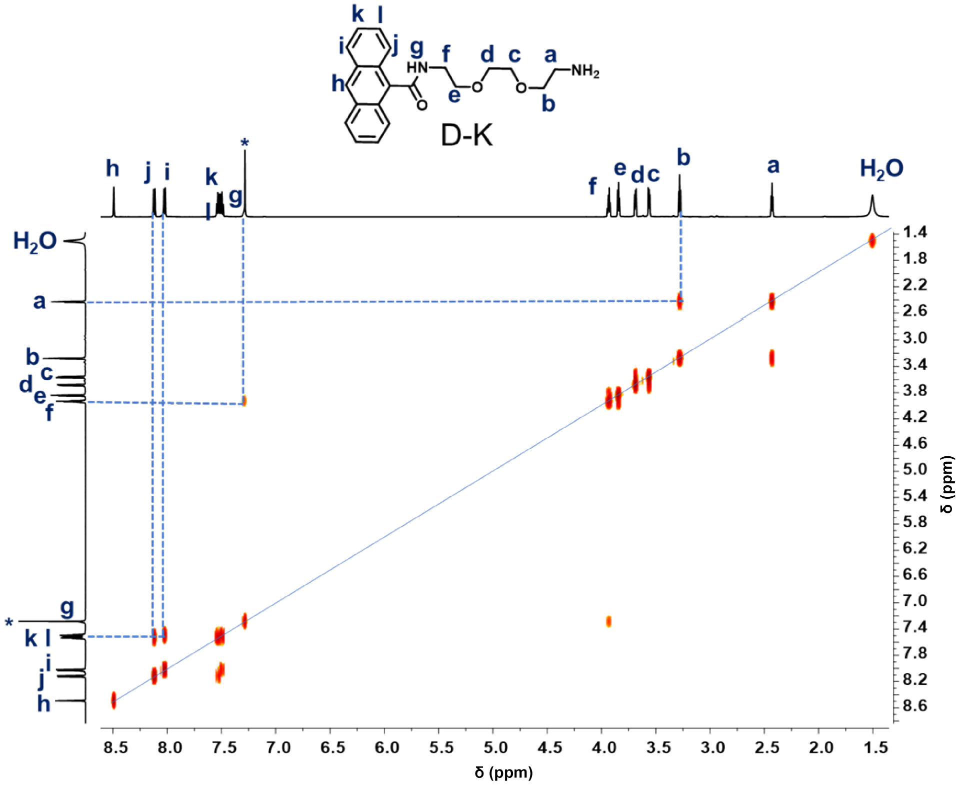

Figure S4: ^1H–^1H COSY (500 MHz, $CDCl_3$) spectrum of **D-K**.

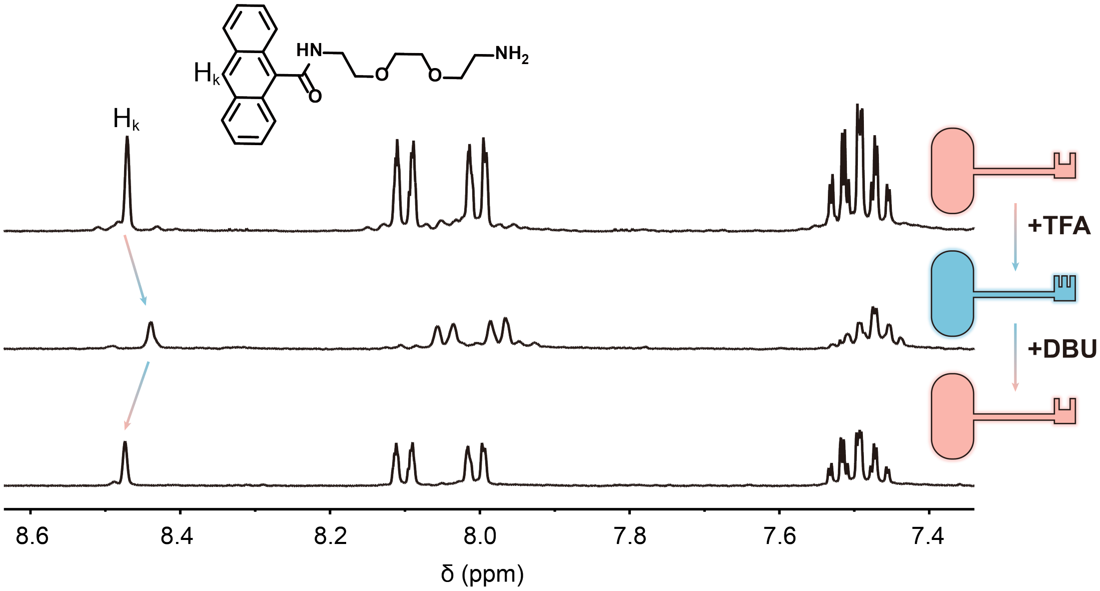

Figure S5: Comparison of the ^{1}H NMR (500 MHz, $CDCl_3$) chemical shift of the H_k proton between **D-K** and **P-K**. Upfield shifts and peak broadening were observed throughout the aromatic region, attributed to some aggregation involving the anthracene moiety induced by protonation in the nonpolar solvent. As an example, the H_k proton exhibited a representative upfield shift and broadening upon conversion from **D-K** to **P-K**. Although such changes in the aromatic region are detectable, the shifts of H_a and the crown ether protons are more distinct and reliable for monitoring the fuel-driven cycling of the system; thus, H_k was not used as the primary indicator in the main text.

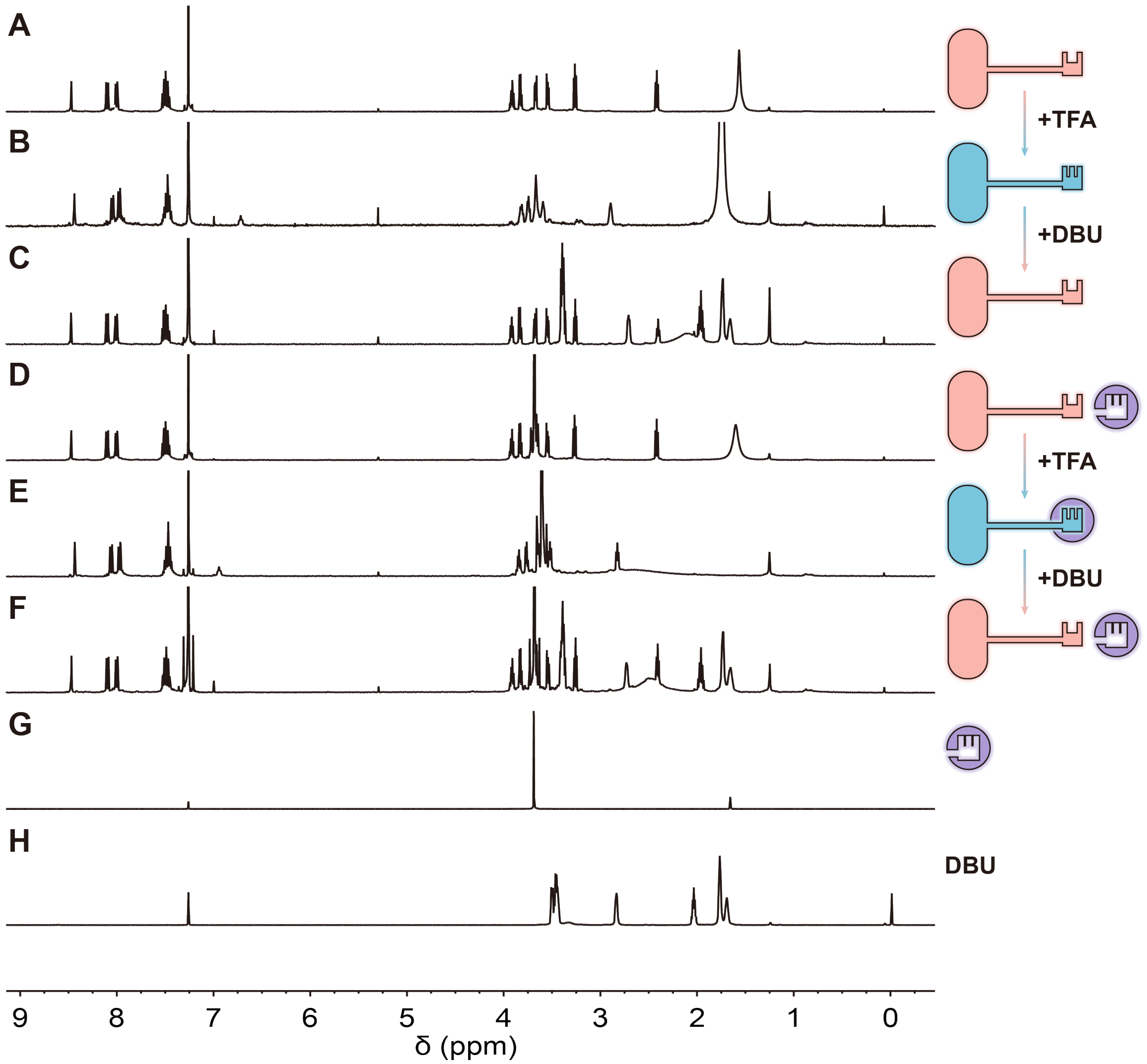

Figure S6: Monitoring each state of the lock-and key system using 1H NMR spectroscopy (Full spectra of Figure. 2). To investigate the protonated and deprotonated states of the key, 3 mM **D-K** was first dissolved in $CDCl_3$ (A), followed by the addition of 3 mM TFA (B), and then an excess of DBU (C). To study the binding and dissociation of the lock and key, 3 mM **L** was dissolved in $CDCl_3$, then 3 mM **D-K** was added (E), followed by 3 mM TFA (F), and finally an excess of DBU (G). (H) shows the ^{1}H NMR spectrum of DBU alone.

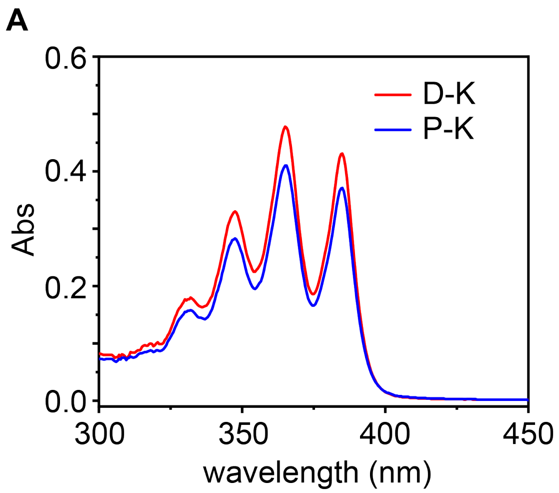

Figure S7: Monitoring the transition from **D-K** to **P-K** using UV-Vis (A) and fluorescence spectroscopy (B).

Figures S8 and S9 present the molecular configurations and electrostatic potential (ESP) distributions of the **D-K** and **P-K** molecules, respectively. As shown in Figure S8, the side chain of the **D-K** molecule forms a certain angle with the conjugated plane of the anthracene core. The van der Waals surface of the molecule shows a negative charge center distributed around the acyl oxygen directly connected to the anthracene core, reflecting its strong electron-withdrawing ability. Partial negative charges are also localized around the nitrogen atoms of the amide and amino groups. Upon protonation to form **P-K**, as seen in Figure S9, the overall stereoconfiguration and molecular skeleton remain largely unchanged. However, the charge distribution undergoes significant alterations. Although the acyl oxygen remains the most negatively charged region, the anthracene core directly connected to it also exhibits noticeable electron distribution after protonation. Additionally, the protonated amino group becomes the most positively charged region of the **P-K** molecule, as indicated by the prominent blue (positive charge) distribution.

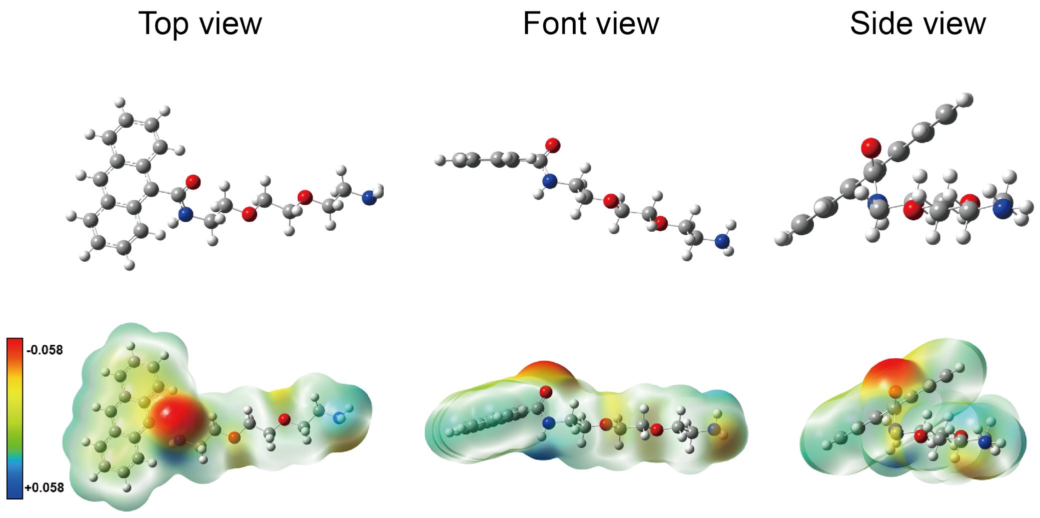

Figure S8: Molecular steric configuration (top) and electrostatic potential (ESP) (bottom) of **D-K** from top view, front view and side view.

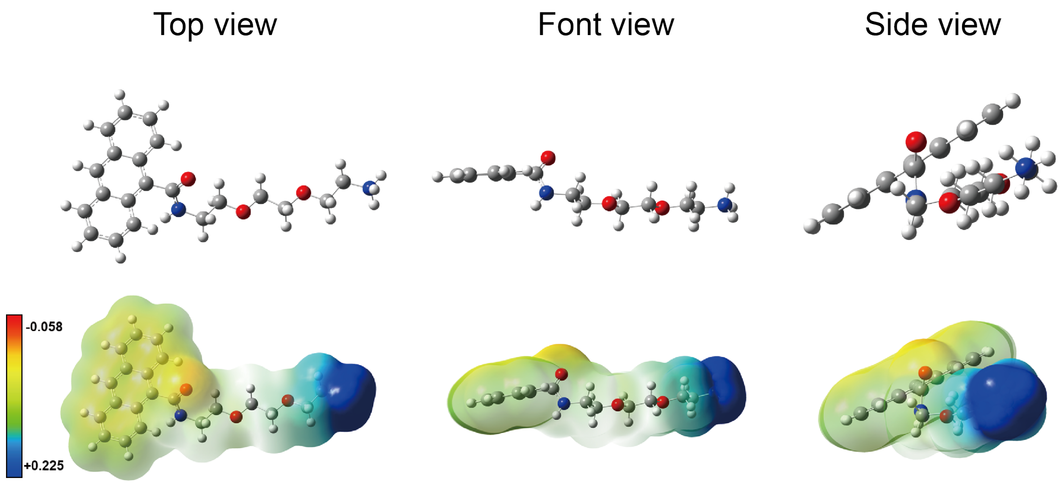

Figure S9: Molecular steric configuration (top) and electrostatic potential (ESP) (bottom) of **P-K** from top view, front view and side view.

Table S1: Energy levels of frontier molecular orbitals for **D-K** and **P-K**.

Molecules	HOMO / eV	LUMO / eV	LUMO+1 / eV
**D-K**	-5.68	-2.22	-0.88
**P-K**	-6.87	-5.38	-3.58

Figure S10 displays the energy calculations based on the optimized molecular configurations, showing the four relevant frontier molecular orbitals (FMOs) of **D-K** and **P-K** , namely the HOMO, LUMO, and LUMO+1. Before protonation, the HOMO, LUMO, and LUMO+1 of the **D-K** molecule are predominantly localized on the conjugated anthracene core, consistent with structures containing similar large conjugated systems, highlighting the electron delocalization capability of the conjugated framework. After protonation, the **P-K** molecule becomes a positively charged system. Consequently, the highly electron-deficient LUMO and LUMO+1 orbitals are localized near the protonated amino group, while the anthracene core, due to its electron-rich conjugated nature, remains the primary region for occupied orbitals such as the HOMO. Correspondingly, the energy levels of the frontier molecular orbitals also shift, as detailed in Table S1.

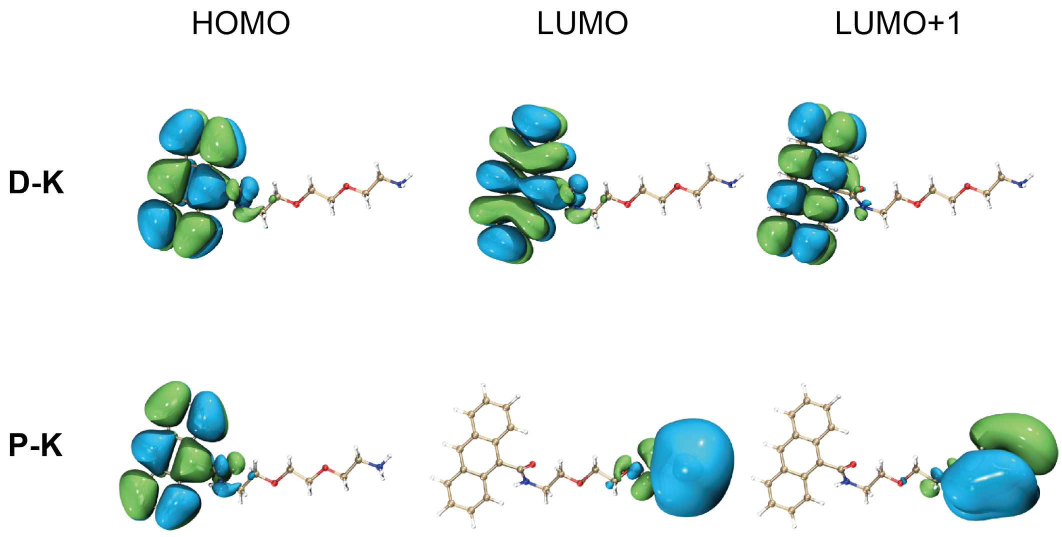

Figure S10: Electronic distribution of frontier molecular orbitals of **D-K** (top) and **P-K** (bottom).

Table S2: Wavelength of UV absorption and FL spectrum peaks for **D-K** and **P-K**.

Molecules	UV max peak / nm	FL peak / nm
**D-K**	358.29	413.37
**P-K**	359.38	419.32

Figure S11 illustrates the electron and hole distributions of the first singlet excited state (S1) for the **D-K** and **P-K** molecules. The white-filled regions represent the spatial origin of the electrons involved in the fluorescence transition, while the gray regions indicate the destination of the electron transition. As shown, before protonation, the fluorescence transition of the **D-K** molecule occurs within the anthracene core itself, representing a localized excitation primarily involving the HOMO-to-LUMO transition. In contrast, after protonation, the fluorescence of the **P-K** molecule arises between the anthracene core and the amino group, indicating a clear charge-transfer excitation involving the transition to the LUMO+1 orbital. Despite the different orbital pairs participating in the excited states of **D-K** and **P-K**, the energy gaps between the HOMO-LUMO of **D-K** and the HOMO-(LUMO+1) of **P-K** are similar, resulting in comparable fluorescence emission peaks and maximum absorption intensities, as summarized in Table S2.

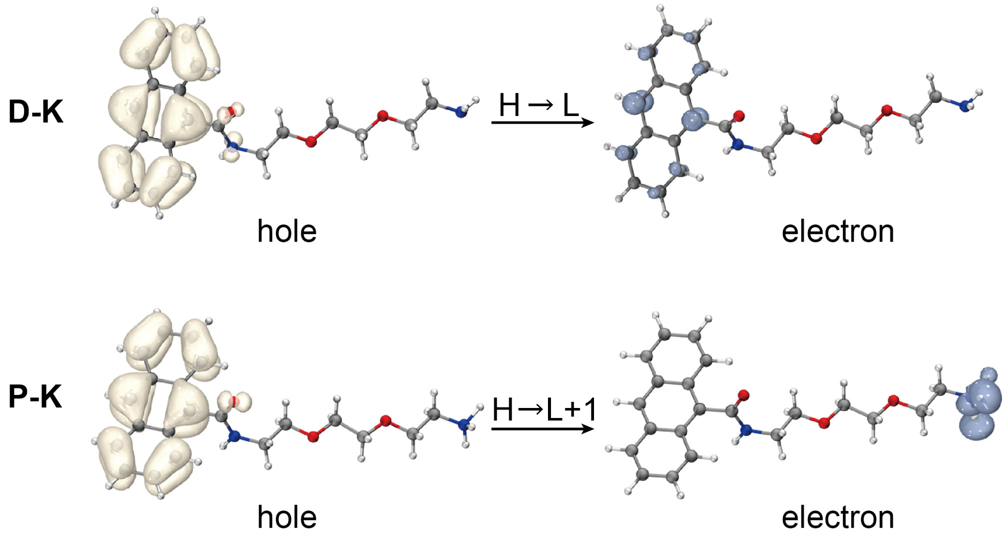

Figure S11: Hole and electron analysis of the first singlet excited states (S1) for **D-K** (top) and **P-K** (bottom), where the involved transition orbitals contributing to the excitation are marked along the arrow.

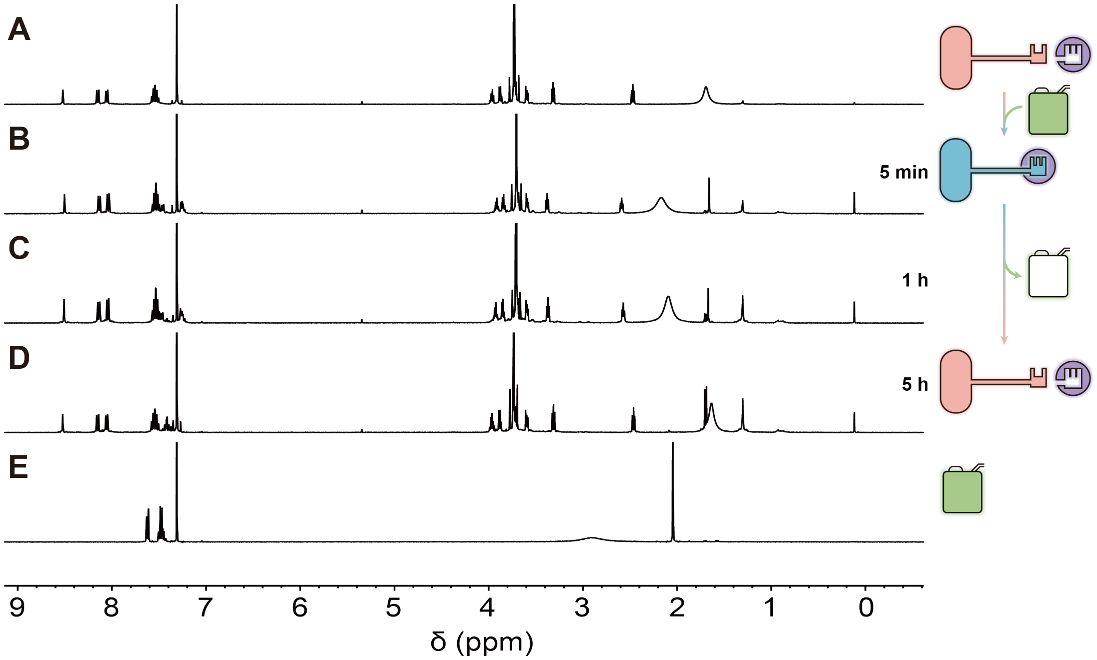

Figure S12: Monitoring the fuel-driven binding between lock and key using ^{1}H NMR spectroscopy (full spectra of Fig. 3A–E). To complete one cycle, 3 mM **D-K** and 3 mM **L** were dissolved in $CDCl_3$ (A), followed by the addition of 3 mM 1 (B). Spectra were recorded after 1 hour (C) and 5 hours (D). (E) shows the ^{1}H NMR spectrum of 1 alone.

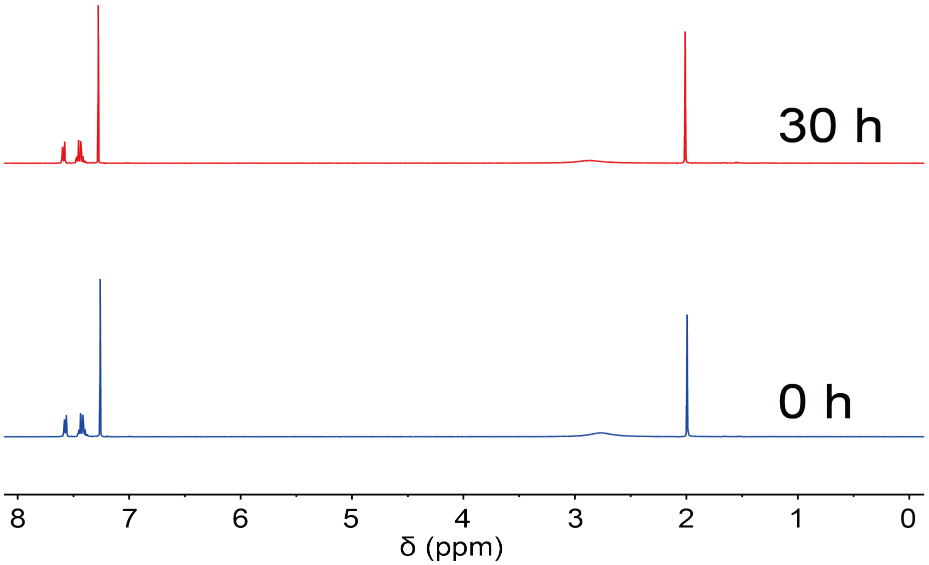

Figure S13: Verification of the stability of chemical fuel **1**. ^{1}H NMR spectra were recorded for a solution of **1** in $CDCl_3$ at 0 hours (blue line) and after 30 hours (red line).

Supplementary References

[1] Becke, A. D. Density-functional thermochemistry. III. The role of exact exchange. *Journal of Chemical Physics* **1993**, *98*, 5648–5652.

[2] Grimme, S.; Ehrlich, S.; Goerigk, L. Effect of the Damping Function in Dispersion Corrected Density Functional Theory. *Journal of Computational Chemistry* **2011**, *32*, 1456–1465.

[3] Petersson, G. A.; Bennett, A.; Tensfeldt, T. G.; Allaham, M. A.; Shirley, W. A.; Mantzaris, J. A complete basis set model chemistry .1. The total energies of closed-shell atoms and hydrides of the 1st-row elements. *Journal of Chemical Physics* **1988**, *89*, 2193–2218.

[4] Weigend, F. Accurate Coulomb-fitting basis sets for H to Rn. *Physical Chemistry Chemical Physics* **2006**, *8*, 1057–1065.

[5] Weigend, F.; Ahlrichs, R. Balanced basis sets of split valence, triple zeta valence and quadruple zeta valence quality for H to Rn: Design and assessment of accuracy. *Physical Chemistry Chemical Physics* **2005**, *7*, 3297–3305.

[6] Lu, T.; Chen, F. Multiwfn: A multifunctional wavefunction analyzer. *Journal of Computational Chemistry* **2012**, *33*, 580–592.

[7] Lu, T. A comprehensive electron wavefunction analysis toolbox for chemists, Multiwfn. *Journal of Chemical Physics* **2024**, *161*.

[8] Humphrey, W.; Dalke, A.; Schulten, K. VMD: visual molecular dynamics. *Journal of molecular graphics* **1996**, *14*, 33–38.

[9] Okuro, K.; Furuune, M.; Miura, M.; Nomura, M. Copper-Catalyzed Reaction of Aryl Iodides with Active Methylene Compounds. *Journal of Organic Chemistry* **1993**, *58*, 7606–7607.
